# Knowledge and Attitude of Rheumatic Fever and Rheumatic Heart Disease Among the Makkah City Population, Saudi Arabia

**DOI:** 10.7759/cureus.51539

**Published:** 2024-01-02

**Authors:** Abdullah Y Fakieha, Dai O Zafer, Safa H Alkalash, Ahmed A Fudah, Rami M Mujlid, Mohammed Y Fakiha, Abdullah Khafajy, Mokhtar M Shatla

**Affiliations:** 1 Department of Cardiology, Al Noor Hospital, Makkah, SAU; 2 Department of Medicine, Umm Al-Qura University, Makkah, SAU; 3 Department of Community Medicine and Healthcare, Umm Al-Qura University, Al-Qunfudah, SAU; 4 Department of Family Medicine, Menoufia University, Shebin El-Kom, EGY; 5 Faculty of Dentistry, Umm Al-Qura University, Makkah, SAU; 6 Ministry of Health, Taif Dental Specialty Center, Taif, SAU; 7 Department of Community Medicine and Healthcare of Pilgrims, Umm Al-Qura University, Makkah, SAU

**Keywords:** group a streptococcal pharyngitis, rheumatic heart, rheumatic fever, attitude, knowledge

## Abstract

Background

Rheumatic heart disease (RHD) is a prominent sequela of rheumatic fever (RF) and the most common cause of acquired valvular disease worldwide. Patients develop RHD as a result of autoimmune reactions caused by an untreated group A Streptococcus (GAS) throat infection, resulting in significant valvular destruction.

Objectives

The current study aimed to assess the knowledge and attitude of RF and RHD among the Makkah city population in Saudi Arabia.

Methods

An observational cross-sectional study was conducted on a convenience sample of 1364 adult participants from Makkah city in Saudi Arabia. Data were collected through an online survey that was disseminated on different electronic platforms. Then, the obtained data were analyzed using SPSS version 23 (IBM Corp., Armonk, New York, USA).

Results

A total of 1364 participants completed the questionnaire; female participants constituted 58.1% (n = 792) and those between 18 and 30 years old represented 57.6% of the sample (n = 785). Knowledge of rheumatic fever was classified as poor (31.9%, n = 435), fair (44.8%, n = 611), and good (23.3%, n = 318). Female participants were shown to have better knowledge than males (p = 0.034). The attitude toward rheumatic fever and rheumatic heart disease was classified as negative (27.9%, n = 380), neutral (49.1%, n = 670), and positive (23%, n = 314).

Conclusions

This study concludes that adults in Makkah city, Saudi Arabia, have poor knowledge about RF and RHD. There is a notable gap in their knowledge regarding the association between sore throat and RF, bacterial dermatitis and RF, the common age for RF, and the necessity of using antibiotics appropriately to prevent this disease. The study also revealed negative attitudes toward RF and RHD among Makkah citizens, but most of them recommended health education campaigns to increase public awareness about this important disease. The results of this study will assist in the development of awareness campaigns about RF and RHD. Finally, qualitative studies are recommended to fully understand what the population perceives about this morbidity.

## Introduction

Acute rheumatic fever (ARF) is a condition due to an immunological reaction to pharyngitis caused by group A Streptococcus (GAS) and Streptococcus pyogenes infections [1]. School age (5-15 years) is the most common age for the development of RF [1]. For the diagnosis of ARF, the revised Jones criteria are employed, which include major criteria such as chorea, erythema marginatum, subcutaneous nodules, polyarthritis, and carditis, while minor criteria involve polyarthralgia, fever, sedimentation rate at or more than 60 mm and/or C-reactive protein (CRP) at or more than 3.0 mg/dl, and prolonged PR interval [2]. To diagnose an initial episode of ARF, two major criteria, or one major plus two minor criteria, are required [2]. Intramuscular injections of benzathine benzylpenicillin G are recommended antibiotics to prevent recurrent ARF attacks [3]. When an individual has a penicillin allergy or adverse effects, macrolide antibiotics are alternatives [4].

Although the number of new cases of acute rheumatic disease is not increasing worldwide, it is still a major cause of cardiovascular disease and the main cause of death [1]. Between 1990 and 2019, the global age-standardized prevalence rate (ASPR) and age-standardized incidence rate of rheumatic heart disease (RHD) increased; by 2030, the ASPR is expected to reach 559.88 per 100,000 people [5]. In 2015, the estimated number of deaths related to RF and its complications was 319,400 [6]. In Saudi Arabia, the percentage of children with RHD is still higher than the worldwide average; the prevalence of RF is 0.3 in 1000, whereas chronic RHD is 2.8 in 1000 [7]. According to a study conducted in Taif, Saudi Arabia, the overall prevalence of RHD among cardiac patients was 8% [8].

The long-term consequences and prevention of RHD and ARF are not well known to the general public. To gradually minimize the burden of RHD, educational activities focused on these themes may prove to be an effective and affordable strategy [9,10]. Health education initiatives can be deployed in many contexts, specifically schools, to increase public knowledge of diseases [10]. Several studies that were conducted in different areas worldwide, including Saudi Arabia, revealed a lack of public awareness of RF and RHD [11-15].

Makkah is the Saudi Arabian holy capital, attracting millions of Muslims from around the world for Hajj and Omrah. In such cramped conditions, respiratory tract illnesses are easily disseminated throughout the population, particularly among Makkah citizens. Because ARF is an immunological consequence of streptococcal infection of the upper respiratory tract, this study was meant to assess the knowledge and attitudes of Makkah city residents toward RF and RHD.

## Materials and methods

Study design

This observational community-based cross-sectional study was executed using a validated self-reported questionnaire, and data collection was completed in a six-month period starting from February to July 2022.

Sample size calculation

In this study, the required sample size was estimated using Epi Info™ 7.1.5 (Center for Disease Control and Prevention; Atlanta, Georgia, USA) based on the entire population of Makkah city (2,015,000) [16] and the frequency of good understanding of ARF from a previous study (41.6%) [17]. Finally, the minimum required sample size is 373.

Study population

Only Makkah city people, male and female, aged 18 and over, who agreed to participate in this study met the inclusion criteria. While the exclusion criteria included those from outside Makkah city, those under the age of 18, and those who did not consent to complete the survey, medical practitioners were excluded through a necessary question at the beginning of the survey about whether or not they are medical practitioners. If the respondents answered yes, they were unable to complete the survey; however, individuals from non-medical professions were able to do so because medical practitioners are not representative of the general population and their responses may have an impact on the study results.

Data collection

Each respondent provided informed consent online before completing a validated questionnaire that was adopted from a recent Saudi study [18]. The questionnaire was then built on a Google Form application and sent to Makkah city residents in an Arabic version between February and July 2022 via social media websites such as Snap Chat, Twitter, and Instagram. To ensure that all collected data came from Makkah city residents, a required question about the resident area was asked, and any submitted answer from anyone other than Makkah citizens would be excluded from the final data analysis. The survey was broken into three sections. The first section inquires about the participants' sociodemographic characteristics. The second section contains nine questions regarding knowledge about RF and RHD, with a score ranging from 0 to 9. Less than 50% was regarded as poor knowledge, 50-75% as fair knowledge, and more than 75% as good understanding. The final component of this survey assessed participants' attitudes about RF and RHD and was graded as less than 50% referring to a negative attitude, 50-75% was considered a neutral attitude, and more than 75% was judged as a positive attitude.

Statistical analysis

SPSS Version 23 (IBM Corp., Armonk, New York, USA) was used to analyze the collected data. The mean and standard deviation were used to describe quantitative data, whereas frequencies and percentages were used for presenting qualitative data. To assess the association between qualitative variables, the chi-squared test (X^2^) was used. When the p-value exceeds 0.05, it is termed "not significant," whereas p-values less than 0.05 are statistically significant.

Ethical approval

The research ethics committee at Umm Al-Qura University, Saudi Arabia, granted ethical permission (HAPO-02-K-012-2022-02-978). The first question on the questionnaire was designed to ensure everyone's agreement while also ensuring the confidentiality of the information acquired.

## Results

Baseline characteristics of the study participants

A total of 1393 responses were submitted over the course of six months. After data filtration, twenty-nine surveys were deleted due to incomplete or invalid responses, with a response rate of 97.9%. Finally, 1364 participants fulfilled the inclusion criteria and completely answered the survey questions. More than half of the respondents were female (58.1%, n = 792). The majority of participants were within the age group of 18 to 30 years (57.6%, n = 785). Regarding monthly income, most of the respondents were earning less than 5000 Saudi Riyal (=1350 $) per month (55.8%, n = 761), and the majority had no children (57.4%, n = 783). Concerning the educational background, 62.4% (n = 851) have university qualifications, and regarding the occupation status, 39.8% (n = 543) were students, while 31.9% (n = 435) were employed (Table 1).

**Table 1:** Sociodemographic characteristics of the study participants (n = 1364). SAR: Saudi Arabian Riyal.

Study data	N = 1364	(%)
Age group		
18–30 years	785	(57.6%)
31–40 years	224	(16.4%)
41–50 years	204	(15.0%)
51–60 years	114	(8.4%)
More than 60 years	37	(2.7%)
Gender		
Male	572	(41.9%)
Female	792	(58.1%)
Monthly income (SAR)		
Less than 5,000	761	(55.8%)
5,000–10,000	268	(19.6%)
10,000–20,000	263	(19.3%)
More than 20,000	72	(5.3%)
Number of children		
None	783	(57.4%)
One to three children	273	(20.0%)
Four to seven children	273	(20.0%)
More than seven children	35	(2.6%)
Educational level		
Secondary	112	(8.2%)
Diploma	152	(11.1%)
University	851	(62.4%)
Postgraduate	249	(18.3%)
Occupational status		
Unemployed	231	(16.9%)
Student	543	(39.8%)
Employed	435	(31.9%)
Retired	102	(7.5%)
Free business	53	(3.9%)

Knowledge regarding rheumatic fever (RF) and rheumatic heart disease (RHD)

A total of 79.6% (n = 1086) of the participants knew that untreated RF can lead to heart disease. A total of 82.1% (n = 1120) of respondents understood that RF is not an infectious disease. Correspondingly, an equivalent level of awareness regarding the characteristics of the disease was also observed. A total of 80% (n = 1091) of the samples were aware that joint pain, inflammation of the heart, rash, and involuntary movement disorder are symptoms of RF. While 46.1% (n = 629) of them knew the association between RF and bacterial dermatitis, 59.1% (n = 806) were aware that sore throats can lead to RF, and 56.5% (n = 771) believed that RF can be prevented if sore throats are managed with antibiotics. The percentage of respondents who thought that antibiotics could be utilized as a preventative measure for heart disease after RF represented 55.1% (n = 752). The percentage of participants who knew that children aged five to 15 were the most susceptible group to developing RF was 45.2% (n = 616). Overall, 73.5% (n = 1003) of the study sample believed that heart disease caused by RF can be cured. Mean (SD) of the overall knowledge was 0.91 (0.73), with a classification of (23.3%, n = 318) of those with good knowledge, (44.8%, n = 611) as fair knowledge, and (31.9%, n = 435) being shown to have poor knowledge towards RF (Table 2).

**Table 2:** The participants' knowledge regarding rheumatic fever (RF) and rheumatic heart disease (RHD) (n = 1364).

Statement	Correct response (N)	(%)
Untreated RF leads to heart disease	1086	(79.6%)
RF is an infectious disease	244	(17.9%)
Joint pain, inflammation of the heart, rash and involuntary movement disorders are symptoms of RF	1091	(80.0%)
There is a relationship between sore throat and RF	806	(59.1%)
Treating sore throat with antibiotics prevents RF	771	(56.5%)
Antibiotics can be used as a preventative method for heart disease after RF	752	(55.1%)
The age group between five and 15 years is less likely to develop RF	748	(54.8%)
There is a relationship between bacterial dermatitis and RF	629	(46.1%)
There is a cure for heart disease caused by RF	1003	(73.5%)
Mean (SD) of the overall knowledge	0.91 (0.73)	
Level of knowledge		
Poor	435	(31.9%)
Fair	611	(44.8%)
Good	318	(23.3%)

The participants' attitudes towards rheumatic fever

A third of the study participants believed that sore throat may be caused by a bacterial or viral infection (33.4%, n = 455), while the majority agreed that doctors are the most appropriate persons to prescribe management of sore throat (90.4%, n = 1233), and antibiotics are the proper method for sore throat management by doctor's prescription (71%, n = 968). Moreover, 61.8% (n = 843) documented that they would visit doctors occasionally whenever their children developed any new symptoms of sore throat, while 8.7% (n = 119) would rather take painkillers than seek medical advice, as about half of them (n = 61 out of 119) perceived that sore throat is a simple condition that does not need medical advice. Most of the participants (76.2%, n = 1040) were unaware of the prophylactic measures for RF. Furthermore, about 46% (n = 628) of the study subjects had a doubt that rheumatic heart disease may occur when rheumatic fever in children is not treated appropriately, and the vast majority (94.7%, n = 1292) recommended public health education programs to raise awareness of RF. The participants' attitudes concerning rheumatic heart disease and rheumatic fever were categorized as neutral (49.1%, n = 670), positive (23%, n = 314), and negative (27.9%, n = 380) (Table 3).

**Table 3:** The participants' attitudes towards rheumatic fever (RF) (n = 1364).

Statement	N = 1364	(%)
Sore throat is a common condition and may be due to		
Cold drinks	258	(18.9%)
Hot drinks	12	(0.9%)
Bacterial or viral infection	455	(33.4%)
All the above	571	(41.9%)
Don’t know	68	(5.0%)
Person who suggested the appropriate treatment for sore throat		
Personal experience	95	(7.0%)
Friend or family	36	(2.6%)
Doctor	1233	(90.4%)
Treatment method for sore throat		
Doctor’s prescription antibiotics	968	(71.0%)
Natural herbs	58	(4.3%)
Honey	126	(9.2%)
Gargling with water and salt	212	(15.5%)
It is important to go to the doctor if your child has sore throat, or do you just take painkillers		
Yes, every time	402	(29.5%)
Yes, sometimes	843	(61.8%)
No, just take painkillers	119	(8.7%)
Rheumatic fever can be prevented		
Yes	324	(23.8%)
No	1040	(76.2%)
Rheumatic heart disease may occur when rheumatic fever in children is not treated appropriately		
Yes	587	(43%)
No	149	(10.9%)
May be	628	(46%)
The creation of health education campaigns to raise awareness about RF is important and recommended		
Yes	1292	(94.7%)
No	72	(5.3%)
Levels of attitude		
Negative	380	(27.9%)
Neutral	670	(49.1%)
Positive	314	(23%)

The sociodemographic factors associated with participants' knowledge about RF and RHD

A chi-squared test was utilized to distinguish the variation in the level of knowledge and its association with the sociodemographic factors of the participants. It showed that female participants possessed a higher level of knowledge than male subjects (p = 0.033). However, there is no prominent variation in the remaining parameters, including age, monthly income, the number of children, and occupational status (p > 0.05) (Table 4).

**Table 4:** Differences in the level of knowledge across the sociodemographic characteristics of the participants (n = 1364). A statistically significant p-value is less than 0.05.

Factors	Level of knowledge							p-value
	Poor (n)	(%)		Fair (n)	(%)	Good (n)	(%)	
Age group								
18-30 years	237	(30.2%)		357	(45.47%)	191	(24.3%)	0.139
31-40 years	66	(29.46%)		108	(48.2%)	50	(22.3%)	
41-50 years	85	(41.6%)		79	(38.7%)	40	(19.6%)	
51-60 years	34	(29.8%)		50	(43.8%)	30	(26.3%)	
More than 60 years	13	(35.1%)		17	(46%)	7	(19%)	
Gender								
Male	199		(34.7%)	233	(40.7%)	140	(24.4%)	0.034
Female	236		(29.8%)	378	(47.7%)	178	(22.4%)	
Monthly income (SAR)								
Less than 5,000	220		(29%)	359	(47%)	182	(24%)	0.067
5,000-10,000	94		(35%)	114	(42.5%)	60	(22.38%)	
10,001-20,000	101		(38.4%)	108	(41%)	54	(20.5%)	
More than 20,000	20		(27.8%)	30	(41.7%)	22	(30.5%)	
Number of children								
None	235		(31.8%)	359	(48.6%)	189	(25.6%)	0.574
One to three children	93		(43%)	123	(45%)	57	(20.87%)	
Four to seven children	93		(34.1%)	114	(41.7%)	66	(24.1%)	
More than seven children	14		(40%)	15	(42.85%)	6	(17.1%)	
Educational level								
Secondary	31		(27.6%)	54	(40.1%)	27	(24.1%)	0.188
Diploma	57		(37.5%)	56	(36.8%)	39	(25.65%)	
University	269		(31.61%)	377	(44.3%)	205	(24%)	
Postgraduate	78		(31.3%)	124	(49.7%)	47	(18.8%)	
Occupational status								
Employed	151		(34.7%)	183	(42%)	101	(23.2%)	0.190
Unemployed	76		(33%)	106	(45.9%)	49	(21.2%)	
Free business	24		(45.2%)	21	(39.6%)	8	(15.1%)	
Student	152		(28%)	254	(46.77%)	137	(25.2%)	
Retired	32		(31.3%)	47	(46.1%)	23	(22.5%)	

## Discussion

Increasing public knowledge and attitude regarding RF and RHD and their degenerative consequences is seen as one of the most crucial steps in preventing catastrophic complications like recurrent attacks of rheumatic fever, valvular lesions, arrhythmias, stroke, and heart failure [19]. Therefore, this study was conducted among 1364 adults from Makkah residents in Saudi Arabia. The results showed that participants' knowledge about RF and RHD was low. Only 23.3% (n = 318) had a good understanding of RF and RHD, whereas 44.8% (n = 611) had fair knowledge and 31.9% (n = 435) had poor knowledge. This outcome is similar to the results of previously conducted studies [15,20]. Kamal et al. [15] conducted a survey-based study targeting people from different Saudi Arabian regions, and they found that about 30% of their sample had poor knowledge about ARF. Additionally, a study carried out in various departments of the Faisalabad Institute of Cardiology, Pakistan, evaluated patients' awareness of various aspects of RHD and came to the conclusion that patients are not well-informed about the disease and its available treatments [20]. These findings raise our attention to the need to conduct comprehensive educational courses for all populations, including patients, about ARF, its complications for the patient's heart, and measures to protect themselves from it and its consequences.

Approximately 79% (n = 1068) of the study subjects knew that RF leads to cardiac complications, and 80% (n = 1091) reported that joint pain, inflammation of the heart, rash, and involuntary movement are the clinical manifestations of RF. There’s an association between sore throat and RF according to 59.1% (n = 806) of the sample, which is a high percentage in comparison to other studies in Cameroon, Pakistan, and Egypt [9,20,21], as this information was known by about 5-34% of these study subjects. A case-control study was carried out in New Zealand on parents whose children had been diagnosed with definite, probable, or possible/borderline rheumatic heart disease (RHD) as a component of the recently implemented school-based RHD echocardiogram screens. The majority of respondents claimed that sore throat is a factor in ARF; case respondents were more likely than control respondents to believe this (78% vs. 65%) (p = 0.033). A significant portion of respondents (89%) and control respondents (83%) agreed that getting a child to see a doctor or nurse as soon as possible is the best course of action when they have sore throat [22].

Management of sore throat with antibiotics is protective against RF, as reported by 56.5% (n = 771) of the study sample; 55.1% (n = 752) answered that antibiotics could be utilized as a preventive medication for the cardiac involvements following RF. These data are significantly higher than the findings of a study by Mougrabi et al. [17], who found that only 5.5% and 5.2% of their study sample, respectively, knew about medical and surgical approaches to RHD management; 19.2% knew that utilizing antibiotics is the primary prevention; and 25.3% and 11.6% knew about the secondary prevention of RHD. Additionally, Almadhi et al. recorded that only 16.2% of their study sample knew that antibiotics can protect against heart complications of RF [19]. These variations in findings between the current and the other two studies could be due to differences in participants' characteristics and tools for data collection.

More than half of the study sample (54.8%, n = 748) knew that children aged from five to 15 years old are the most susceptible population to developing RF. The association between bacterial dermatitis and RF was recognized by 46.1% (n = 629), while 73.5% (n = 1003) of respondents knew that RHD can be managed. In contrast with the findings of another Saudi study, 33% of the study sample did not know that untreated RF can lead to heart diseases; only 12.4% determined that RF is more likely to occur in children aged 5-15 years; and only 9.7% recognized the link between RF and bacterial dermatitis [19].

Approximately 95% (n = 1292) of the study subjects sought awareness campaigns for RF. Correspondingly with Almadhi et al., 91.2% of their study sample supports the importance of attending health education sessions about RHD [19]. Also, Sayed et al. [21] remarked that health education helps raise public understanding about the causes and treatment of RHD. Additionally, a previously done Saudi study proved that an educational childhood and adolescence safety campaign for caregivers resulted in a significant increase in the overall knowledge and attitudes toward childhood and adolescence safety [23]. All the previously mentioned data underscores the necessity of educating the public about ARF and RHD through organized campaigns.

According to the current study findings, gender was the only significant factor affecting knowledge of RF and RHD among Makkah citizens. In particular, females have better knowledge than males, which corresponds to the results of Mougrabi et al. and Sayed et al. [17,21]. Female participants scored better because they are more likely to be caregivers and more aware of child health issues.

Approximately half (49.1%, n = 670) of this study's participants had neutral attitudes toward ARF and RHD; only 23.4% (n = 314) were positive toward RF and RHD, while 27.9% (n = 380) were negative. Furthermore, 41.9% (n = 571) of participants believed that cold and hot liquids as well as viral and bacterial illnesses were causes of sore throat development. In the Almadhi et al. study, 53.1% of the study participants believed that bacterial and viral infections caused sore throats [19], while Nkoke et al. discovered that 73.4% of their study subjects were unaware of the reasons for sore throats [9]. Causes and adequate management of RF need to be accurately addressed in the health education sessions for the public to protect themselves and their children from the occurrence of sore throat, which is considered the gateway for streptococcal infection and its complications.

In this study, 90.4% (n = 1223) of participants determined that only doctors are capable of recommending the correct treatment for sore throat, and 71% (n = 968) reported that doctors can also prescribe suitable antibiotics for patients with sore throat. Nkoke et al. [9] discovered that, whereas 71.1% of participants in their study reported having sore throats, only 45.4% of those cases were treated with antibiotics that were prescribed by doctors in 35.8% of the cases [9]. Also, an Italian study evaluated individuals from 13 different nations who reported having sore throats in the preceding year and revealed that 30% of the study sample consulted general practitioners to manage sore throats [24]. When comparing the current study findings with those from the other two studies, the current findings are better as most study subjects do not rely on self-medication for sore throat management.

In summary, "Key findings: 31.9% had poor knowledge, 44.8% had fair knowledge, and 23.3% had good knowledge about RF/RHD. Females had better knowledge compared to males; 76% were unaware that RF can be prevented; and 94.7% agreed campaigns are needed to raise awareness." We suspect that poor knowledge and attitudes about RF and RHD could be attributed to the fact that most cases of sore throats are caused by viral and other non-bacterial causes, so healthcare providers may ignore the need to educate patients about RF and its complications.

Limitations and strengths of the study

Limitations of this study include the reliance on self-reported survey data, which may be susceptible to convenience sampling bias, potentially excluding individuals who do not use social media. To address such limitations, future research could benefit from using interviewer-administered questionnaires to enhance population coverage and gather more diverse perspectives. The study did not ask about the source of knowledge about RF and RHD; therefore, we are unable to predict whether they received adequate information about this health issue from the healthcare providers or not, particularly since most participants recommended going to health education events to learn more about this serious illness. Despite the previously mentioned limitations, this study is the first that has been done in the major holy city of Makkah, with a high rate of international visitors to Hajj and Omrah. It is considered an initiative for further research to completely understand how the public views RF and RHD and the role that healthcare providers play in teaching the public about these diseases. Qualitative research is recommended to provide deep insight about RF and RHD among people.

## Conclusions

The study concludes that there is poor knowledge about RF and RHD among the people living in Makkah city, Saudi Arabia. It is surprising how people did not know enough about the relationships between RF and sore throat, between RF and bacterial dermatitis, the common age of RF, and the need for timely and appropriate antibiotic administration to prevent this illness. Unfortunately, the study population perceived RF and RHD negatively. Healthcare providers, especially family physicians, who are the most closely in contact with the community population, should take the initiative to educate people attending primary care centers (PHCs) about RF and its complications, specifically patients who attend PHCs complaining of sore throats and those having tonsillitis, and to counsel them to be compliant with the management plan with the prescribed antibiotics without misuse. It is recommended that policymakers create health education programs for Makkah's population either through in-person or online attendance. Information about the etiology, pathophysiology, risk factors, clinical picture, and preventative measures for RF and RHD should be explained in these sessions. Finally, it is advised to conduct a study to assess the level of preparedness among medical professionals, particularly family physicians and their teams in PHCs, to educate PHCs' attendees about RF and RHD.

## Appendices

Questionnaire : Knowledge and Attitude of Rheumatic Fever and Rheumatic Heart Disease among Makkah City Population, Saudi Arabia

 Are you willing to take part in the study, knowing that no identifying personal information about the participants will be published?

• Yes

• No

 **Are you a medical practitioner?**

• Yes

• No 

Sociodemographic characteristics of the participants

Age group in years

·       18-30

·       31-40

·       41-50

·       51-60

·       More than 60 years

Gender

·       Male

·       Female

Residence region

·       Makkah city

·       Outside Makkah city

**Monthly income (SAR)**
 

·       Less than 5,000

·       5,000 to 10,000

·       10,001 to 20,000

·       More than 20,000

Number of children

·       None

·       1 to 3 children

·       4 to 7 children

·       More than 7 children

Educational level

·       Primary

·       Secondary

·       Diploma

·       University

·       Postgraduate

Occupational status

·       Unemployed

·       Student

·       Employed

·       Retired

·       Free business

     Assessment of the knowledge regarding rheumatic fever (RF).

1.     Does untreated RF lead to heart disease?                                         (True / False)

2.     Is RF an infectious disease?                                                             (True / False)

3.     Is improving housing and living standards a preventive measure that helps reduce the incidence of RF?    (True / False)

4.     Are joint pain, inflammation of the heart, rash and involuntary movement disorders symptoms of RF?         (True / False)

5.     Is there a relationship between sore throat and RF?                        (True / False)

6.     Does treating sore throat with antibiotics prevent RF?                   (True / False)

7.     Can antibiotics be used as a preventative treatment for heart disease after RF?              (True / False)

8.     Is The age group between 5 and 15 years less likely to develop RF?       (True / False)

9.     Is there a relationship between bacterial dermatitis and RF?                     (True / False)

10.  Is there a cure for heart disease caused by RF?                                          (True / False)

Attitudes about rheumatic fever (RF) and its treatment

Which of the following may be causes of sore throat?

·       Cold drinks

·       Cold weather

·       Bacterial or viral infection

·       All the above

·       I don’t know

**Which one of the following is the person who could suggest the appropriate treatment of sore throat?**

·       Personal experience

·       Friend or family

·       Doctors

Which one of the following is the treatment method for sore throat?

·       Doctor’s prescription antibiotics

·       Natural herbs

·       Honey

·       Gargling with water and salt

**Do you think it is important to go to the doctor if your child has sore throat, or do you just take painkillers?**
 

·       Yes, every time

·       Yes, sometimes

·       No, just take painkillers

If no, state the reason

·       Lack of time

·       There is no need

·       Other

Can rheumatic fever be prevented?

·       Yes

·       No

**May rheumatic heart disease occur when rheumatic fever in children is not treated appropriately?**

·       Yes

·       No

·       May be

Do you support the creation of campaigns to raise awareness about RF?

·       Yes

·       No

·       Maybe

 استبيان :المعرفة والموقف من الحمى الروماتيزمية وأمراض القلب الروماتيزمية بين سكان مدينة مكة المكرمة، المملكة العربية السعودية

هل ترغب بالمشاركة في الدراسة مع التأكيد من جانبنا على عدم نشر أي معلومات شخصية تخص المشاركين؟

·       نعم

·       لا

هل انت ممارس طبي؟

·       نعم

·       لا

ل**معلومات الديموجرافية****:**

ا**لعمر:**

·       ١٨-٣٠ سنه

·       ٣١- ٤٠ سنه

·       ٤١- ٥٠ سنه

·       ٥١-٦٠ سنه

·       اكثر من ٦٠ سنه

ا**لجنس:**

·       ذكر

·       انثى

السكن

·       مدينة مكة المكرمة

·       من خارج مدينة مكة المكرمة

الدخل الشهري

·       اقل من٥٠٠٠ ريال سعودي

·       ٥٠٠٠ الى ١٠٠٠٠ ريال سعودي

·       ١٠٠٠١ الى ٢٠٠٠٠ ريال سعودي

·       اكثر من ٢٠٠٠٠ ريال سعودي

عدد الأطفال

·       لا يوجد

·       ١-٣ أطفال

·       ٤-٧ أطفال

·       اكثر من ٧ أطفال

المؤهل التعليمي

·       إبتدائي

·       ثانوي

·       دبلوم

·       جامعي

·       دراسات عليا

الوضع المهني

·       غير موظف

·       طالب

·       موظف

·       متقاعد

·       اعمال حره

     **تقييم معرفه مرض الحمى الروماتيزمية**

1.     هل عدم معالجة الحمى الروماتيزمية يؤدي لمرض في القلب؟                             (صح/خطآ)

2.     هل الحمى الروماتيزمية هو مرض معدي؟                                                    (صح/خطآ)

3.     هل يعد تحسين مستويات السكن والمعيشة إجراءً وقائيًا يساعد في تقليل حالات الإصابة بالحمى الروماتيزمية؟ (صح/خطآ)

4.     هل من أعراض الحمى الروماتيزمية (آلام المفاصل والتهاب القلب والطفح الجلدي واضطرابات الحركة اللاإرادية) ؟                        (صح/خطآ)

5.     هل هناك علاقة بين التهاب الحلق والحمى الروماتيزمية؟                                (صح/خطآ)

6.     هل علاج التهاب الحلق بالمضادات الحيوية يمنع الإصابة بالحمى الروماتيزمية؟   (صح/خطآ)

7.     هل يمكن استخدام المضادات الحيوية كعلاج وقائي لأمراض القلب بعد الإصابة بالحمى الروماتيزمية؟ (صح/خطآ)

8.     هل الفئة العمرية ما بين 5 إلى 15 سنة أقل عرضة للإصابة بالحمى الروماتيزمية؟      (صح/خطآ)

9.     هل هناك علاقة بين التهاب الجلد البكتيري والإصابة بالحمى الروماتيزمية؟               (صح/خطآ)

10.  هل هناك علاج لأمراض القلب الناتجة عن الإصابة بالحمى الروماتيزمية؟                (صح/خطآ)

   **  السلوك اتجاه الحمى الروماتيزمية **

ما هي أسباب الإصابة بـالتهاب الحلق؟

·       مشروبات باردة

·       طقس بارد

·       عدوى بكتيرية أو فيروسية

·       جميع ما سبق

·       لا أعلم

**من هو الشخص الذي يقترح العلاج المناسب لالتهاب الحلق**؟

·       خبرة شخصية

·       الأصحاب او الاهل

·       الطبيب

ماهي طريقة علاج التهاب الحلق؟

·       المضادات الحيوية التي يصفها الطبيب

·       أعشاب طبيعية

·       عسل

·       الغرغرة بالماء والملح

هل تعتقد أنه من المهم الذهاب إلى الطبيب إذا كان طفلك يعاني من التهاب في الحلق، أم أنك تعطيه مسكنات الألم فقط؟

·       نعم، في كل مره

·       نعم، أحيانا

·       لا، اكتفي بمسكنات الألم

إذا كان جوابك لا، الرجاء تحديد السبب

·       قلة الوقت

·       لا حاجة للذهاب للطبيب

·       غير ذلك

هل يمكن الوقاية من الحمى الروماتيزمية؟

·       نعم

·       لا

هل الحمى الروماتيزمية قد تسبب أمراض القلب اذا لم يتم علاجها؟

·       نعم

·       لا

·       احتمال

هل تؤيد إنشاء حملات لرفع مستوى الوعي حول الحمى الروماتيزمية؟

·       نعم

·       لا

·       احتمال

## References

[1] Carapetis JR, Beaton A, Cunningham MW (2016). Acute rheumatic fever and rheumatic heart disease. Nat Rev Dis Primers.

[2] Gewitz MH, Baltimore RS, Tani LY (2015). Revision of the Jones Criteria for the diagnosis of acute rheumatic fever in the era of Doppler echocardiography: a scientific statement from the American Heart Association. Circulation.

[3] Beaton A, Okello E, Rwebembera J (2022). Secondary antibiotic prophylaxis for latent rheumatic heart disease. N Engl J Med.

[4] Ralph AP, Currie BJ (2022). Therapeutics for rheumatic fever and rheumatic heart disease. Aust Prescr.

[5] Hu Y, Tong Z, Huang X (2022). The projections of global and regional rheumatic heart disease burden from 2020 to 2030. Front Cardiovasc Med.

[6] Watkins DA, Johnson CO, Colquhoun SM (2017). Global, regional, and national burden of rheumatic heart disease, 1990-2015. N Engl J Med.

[7] Seckeler MD, Hoke TR (2011). The worldwide epidemiology of acute rheumatic fever and rheumatic heart disease. Clin Epidemiol.

[8] Algethami A Algethami A,  Althobaiti O,  Althomali I (2018). Prevalence of rheumatic heart disease and its risk factors among cardiac patients in Taif City, KSA. Egyptian J Hosp.

[9] Nkoke C, Luchuo EB, Jingi AM, Makoge C, Hamadou B, Dzudie A (2018). Rheumatic heart disease awareness in the South West region of Cameroon: a hospital based survey in a Sub-Saharan African setting. PLoS One.

[10] Oliveira KK, Nascimento BR, Beaton AZ (2020). Health education about rheumatic heart disease: a community-based cluster randomized trial: rheumatic heart disease educational strategies. Glob Heart.

[11] Prasad A, Prasad A, Singh BK, Kumar S (2020). Compliance to the secondary prophylaxis and awareness of rheumatic heart disease: a cross-sectional study in low-income province of India. J Fam Med Prim Care.

[12] Regmi PR, Sanjel K (2019). Effectiveness of awareness raising interventions on knowledge about rheumatic heart disease and change in care seeking behavior for throat infection in Lalitpur, Nepal. Nepal Heart J.

[13] Lawrence JG, Carapetis JR, Griffiths K, Edwards K, Condon JR (2013). Acute rheumatic fever and rheumatic heart disease: incidence and progression in the Northern Territory of Australia, 1997 to 2010. Circulation.

[14] Abdul-Mohsen MF, Lardhi AA (2011). A dramatic decline in university hospital admissions of acute rheumatic fever in the eastern region of Saudi Arabia. J Saudi Heart Assoc.

[15] Kamal SM, Hotan ASB, Alanzan AAA (2019). Knowledge, awareness, and attitude of Saudi population toward rheumatic fever. IJMDC.

[16] (2023). Saudi Arabia (KSA) Population Statistics 2023 (Infographics). https://www.globalmediainsight.com/blog/saudi-arabia-population-statistics/.

[17] Mougrabi MM, Aljuaid RS, Alrabie AD, Althumali NK, Alkhaldi LH, Alotaibi WD (2021). Awareness of rheumatic fever and rheumatic heart disease among the population in Taif, Saudi Arabia 2020. J Family Med Prim Care.

[18] ElTellawy M, Alfallaj M, Aldaghmi R (2021). Parents’ knowledge and attitudes toward rheumatic heart disease in Saudi Arabia. Int J Med Dev Countries.

[19] Almadhi AA, Alshammri MR, Altamimi NO, Hadal SA, Al Madhi AA, Salahie MS (2021). Rheumatic fever and rheumatic heart disease-related knowledge, attitude, and practice in Saudi Arabia. Cureus.

[20] Saeed M, Afzal M (2016). Awareness of rheumatic heart disease in patients suffering from rheumatic heart disease. J University Med Dent Coll.

[21] Sayed AK, Se'eda H, Eltewacy NK (2021). Awareness of rheumatic heart disease in Egypt: a national multicenter study. J Cardiovasc Dev Dis.

[22] Gurney JK, Chong A, Culliford-Semmens N, Tilton E, Wilson NJ, Sarfati D (2017). High levels of rheumatic fever and sore throat awareness among a high-risk population screened for rheumatic heart disease. N Z Med J.

[23] Temsah MH, Aljamaan F, Alhaboob A (2022). Enhancing parental knowledge of childhood and adolescence safety: an interventional educational campaign. Medicine (Baltimore).

[24] van der Velden AW, Sessa A, Altiner A, Pignatari AC, Shephard A (2020). Patients with sore throat: a survey of self-management and healthcare-seeking behavior in 13 countries worldwide. Pragmat Obs Res.

